# The chaperone GRP78 is a host auxiliary factor for SARS-CoV-2 and GRP78 depleting antibody blocks viral entry and infection

**DOI:** 10.1016/j.jbc.2021.100759

**Published:** 2021-05-07

**Authors:** Anthony J. Carlos, Dat P. Ha, Da-Wei Yeh, Richard Van Krieken, Chun-Chih Tseng, Pu Zhang, Parkash Gill, Keigo Machida, Amy S. Lee

**Affiliations:** 1 Department of Biochemistry and Molecular Medicine, Keck School of Medicine, University of Southern California, Los Angeles, California, USA; 2 USC Norris Comprehensive Cancer Center, Keck School of Medicine, University of Southern California, Los Angeles, California, USA; 3 Department of Molecular Microbiology and Immunology, Keck School of Medicine, University of Southern California, Los Angeles, California, USA; 4 Department of Medicine, Keck School of Medicine, University of Southern California, Los Angeles, California, USA

**Keywords:** SARS-CoV-2, GRP78, Spike protein, ACE2, antibody, ACE2, angiotensin-converting enzyme 2, GRP78, 78-kDa glucose-regulated protein, hMAb159, humanized monoclonal antibody, RBD, receptor-binding domain, SARS-CoV-2, severe acute respiratory syndrome coronavirus 2, SARS-2-S, SARS-CoV-2-Spike protein, VeroE6-ACE2, VeroE6 cells overexpressing ACE2

## Abstract

The severe acute respiratory syndrome coronavirus 2 (SARS-CoV-2), the causative agent of the COVID-19 global pandemic, utilizes the host receptor angiotensin-converting enzyme 2 (ACE2) for viral entry. However, other host factors might also play important roles in SARS-CoV-2 infection, providing new directions for antiviral treatments. GRP78 is a stress-inducible chaperone important for entry and infectivity for many viruses. Recent molecular docking analyses revealed putative interaction between GRP78 and the receptor-binding domain (RBD) of the SARS-CoV-2 Spike protein (SARS-2-S). Here we report that GRP78 can form a complex with SARS-2-S and ACE2 on the surface and at the perinuclear region typical of the endoplasmic reticulum in VeroE6-ACE2 cells and that the substrate-binding domain of GRP78 is critical for this interaction. *In vitro* binding studies further confirmed that GRP78 can directly bind to the RBD of SARS-2-S and ACE2. To investigate the role of GRP78 in this complex, we knocked down GRP78 in VeroE6-ACE2 cells. Loss of GRP78 markedly reduced cell surface ACE2 expression and led to activation of markers of the unfolded protein response. Treatment of lung epithelial cells with a humanized monoclonal antibody (hMAb159) selected for its safe clinical profile in preclinical models depleted cell surface GRP78 and reduced cell surface ACE2 expression, as well as SARS-2-S-driven viral entry and SARS-CoV-2 infection *in vitro*. Our data suggest that GRP78 is an important host auxiliary factor for SARS-CoV-2 entry and infection and a potential target to combat this novel pathogen and other viruses that utilize GRP78 in combination therapy.

The coronavirus pandemic caused by the severe acute respiratory syndrome coronavirus 2 (SARS-CoV-2) is currently the greatest threat to global public health. While SARS-CoV-2 vaccines provide optimism to combat COVID-19, identification of targets that may offer therapy for those ineligible for vaccine or infected by escape mutants bypassing vaccine protection is of great interest. While it has been elucidated that the SARS-CoV-2-Spike protein (SARS-2-S) responsible for viral attachment and fusion to the host cells exploits angiotensin-converting enzyme 2 (ACE2) as the cellular receptor for viral entry, evidence is emerging that other host factors may serve as critical entry cofactors for productive infection (1, 2). Recent molecular docking analyses have identified a putative site of interaction between the 78 kilo-Dalton glucose-regulated protein (GRP78) and the receptor-binding domain (RBD) of SARS-2-S, raising the possibility that GRP78 can facilitate or serve as an alternative receptor for SARS-CoV-2 entry (3, 4). Furthermore, computer modeling reveals that host-cell recognition through GRP78 is enhanced in the new UK variant of SARS-CoV-2 associated with increased transmissibility, as well as in the emerging 501.V2 South African variant (5, 6).

GRP78, also known as BiP and encoded by the *HSPA5* gene, is the major HSP70 family member in the endoplasmic reticulum (ER) serving critical protein folding functions (7, 8). In addition, GRP78 is a master regulator of the unfolded protein response, which allows cells to adapt to adverse stress conditions targeting the ER (9, 10, 11). GRP78 is broadly expressed in many tissues including bronchial epithelial cells and the respiratory mucosa at levels significantly higher than that of ACE2 (12). In recent case-control studies, serum GRP78 levels were found to be elevated in SARS-CoV-2 cases (13). Under pathophysiological conditions such as cancer and viral infection, GRP78 can translocate from the ER to the cell surface where it acts as a coreceptor for various signaling molecules, as well as for viral entry (10, 14, 15, 16, 17, 18, 19, 20, 21). For coronaviruses, GRP78 is known to interact with the bat coronavirus HKU9 and MERS-CoV Spike proteins, facilitating cell surface attachment and viral entry (22). Furthermore, virus infection leads to ER stress and increased total and cell surface GRP78 (csGRP78) expression further enhancing viral infection in a positive feedback cycle (15, 22). Here, utilizing biochemical and imaging approaches, we established GRP78 interactions with SARS-2-S and ACE2. We further demonstrated that a humanized monoclonal antibody (hMAb159) with high affinity and specificity against GRP78 and a safe clinical profile in preclinical models (23) depletes csGRP78 and reduces cell surface ACE2 (csACE2), SARS-CoV-2 entry, and infection.

## Results

### GRP78 forms complex with SARS-CoV-2 Spike protein and host receptor ACE2

**Figure 1:**
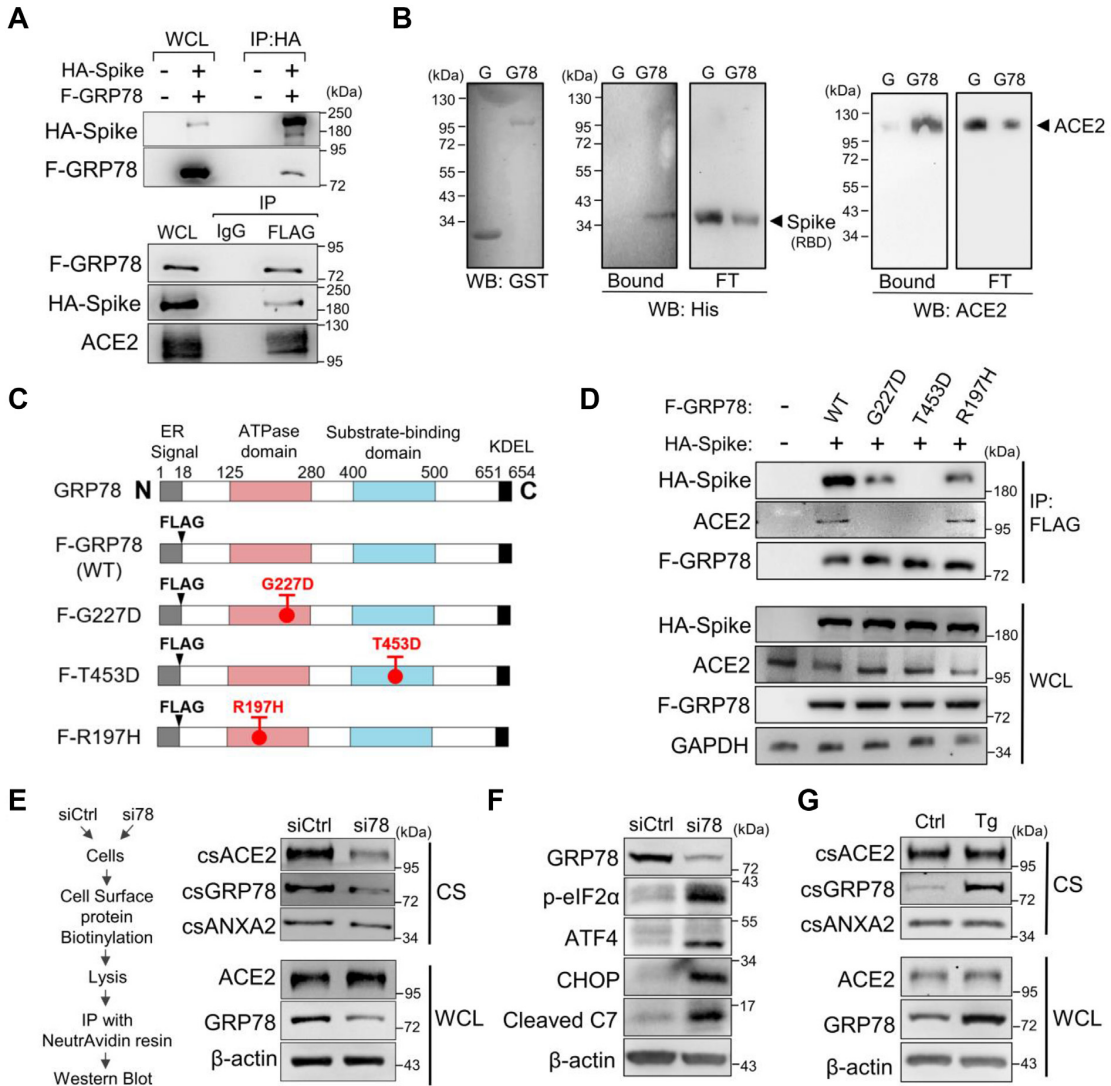
**GRP78 interacts with SARS-CoV-2 Spike protein and ACE2.***A*, lysates from VeroE6-ACE2 cells expressing FLAG-GRP78 (F-GRP78) and HA-Spike were subjected to immunoprecipitation (IP) using the anti-HA, IgG, or anti-FLAG antibodies as indicated. The whole cell lysate (WCL) and IP fractions were analyzed for the indicated proteins by western blot, using anti-HA for detection of HA-Spike and anti-FLAG antibody for F-GRP78. *B*, western blot analysis of *in vitro* binding assays. GST (G) or GST-tagged GRP78 (G78) proteins affixed to Glutathione Sepharose resin were incubated with His-tagged recombinant SARS-CoV-2 Spike receptor-binding domain (RBD) or His-tagged recombinant human ACE2 protein. The input GST and G78 proteins, the bound and flow through (FT) fractions showing unbound proteins were subjected to western blot using antibodies against GST, His, or ACE2 as indicated. *C*, schematic illustration of the indicated domains of GRP78 and FLAG-tagged wild-type (WT) and mutated forms of human GRP78 encoded by the expression plasmids. *D*, similar to *A* except lysates from cells expressing WT or the indicated mutant forms of GRP78 were subjected to IP with anti-FLAG antibody, with GADPH serving as loading control for the WCL. *E*, WCL or purified biotinylated cell surface (CS) proteins isolated in the indicated experimental scheme were probed for GRP78 and ACE2 levels by western blot. β-actin served as loading control for WCL and Annexin A2 (ANXA2) for CS proteins. *F*, VeroE6-ACE2 cells were treated with either control siRNA (siCtrl) or siRNA against GRP78 (si78) and lysates were probed for the indicated markers of the unfolded protein response and cleaved caspase 7 (C7) by western blot. *G*, western blot analysis of GRP78 and ACE2 levels in biotinylated cell surface (CS) fraction or WCL in VeroE6-ACE2 cells following treatment of 300 nM of ER stress inducer thapsigargin (Tg) for 24 h. ANXA2 served as loading control for CS and β-actin for WCL.

To test GRP78 binding to SARS-2-S in cells, we expressed HA-tagged SARS-2-S (HA-Spike) and FLAG-tagged GRP78 (F-GRP78) in African green monkey kidney epithelial VeroE6 cells overexpressing human ACE2 (VeroE6-ACE2) as a model system. Co-immunoprecipitation (IP) for the HA-epitope showed that F-GRP78 can be pulled down with HA-Spike suggesting potential interaction between the two proteins (Fig. 1*A*). Co-IP with an antibody against the FLAG-epitope further showed that F-GRP78 can bind HA-Spike and ACE2 (Fig. 1*A*). Furthermore, GST-GRP78 can bind to recombinant SARS-2-S receptor-binding domain (RBD) as well as recombinant ACE2 in *in vitro* pull-down assays (Fig. 1*B*), suggesting a direct binding interaction between GRP78 and both Spike RBD and ACE2. GRP78 contains an ATP-binding domain required for its ATPase catalytic activity in protein folding and a substrate-binding domain required for interaction with its client proteins (Fig. 1*C*). Utilizing the dominant negative mutant G227D unable to bind ATP, the T453D mutant unable to bind protein substrates and the R197H mutant, which renders GRP78 unable to associate with cochaperone DnaJ proteins (20), we probed whether any of these activities is required for GRP78 binding to SARS-2-S and ACE2. Upon transfection of the FLAG-tagged expression vectors into VeroE6-ACE2 cells, we observed that the WT and three mutant proteins were expressed at similar levels (Fig. 1*D*). Compared with WT GRP78, G227D and R197H mutants bound to HA-Spike, albeit at a lower level, while the T453D mutant did not, whereas both G227D and T453D mutants were unable to bind ACE2 (Fig. 1*D*). Collectively, these results indicate that GRP78 can directly bind to the RBD of SARS-2-S and the SBD of GRP78 is most critical for interaction between GRP78 and SARS-2-S providing experimental evidence consistent with a previous *in silico* molecular docking study (4). Additionally, GRP78 can directly bind ACE2 and that binding to ACE2 requires both the SBD and the ATP-binding domain.

### GRP78 colocalizes with SARS-2-S and ACE2 at the cell surface and the perinuclear region

**Figure 2:**
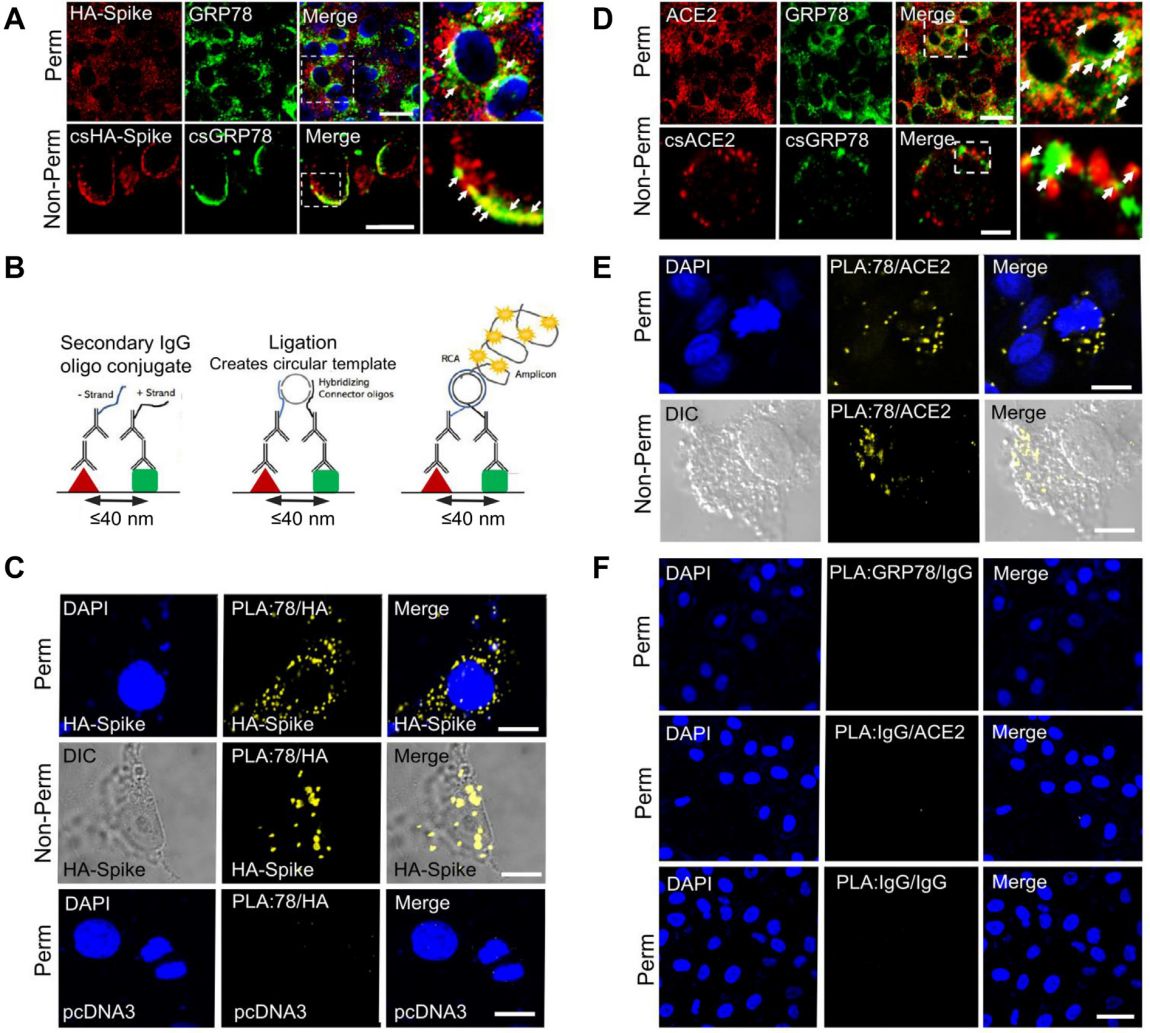
**GRP78 interactions with SARS-CoV-2 Spike protein and ACE2 by confocal immunofluorescence and proximity ligation assay.***A*, confocal immunofluorescence (IF) images of VeroE6-ACE2 cells expressing HA-Spike probed with anti-HA (*red*) and anti-GRP78 (*green*) antibodies. The *top* and *bottom rows* represent permeabilized (Perm) and nonpermeabilized (non-Perm) cells respectively. The *boxed areas* are enlarged on the *right*. *Arrows* indicate costaining. (Scale bars, 20 μm). *B*, schematic diagram of the proximity ligation assay (PLA). *C*, VeroE6-ACE2 cells transfected with vector expressing HA-Spike or empty vector (pcDNA3) as indicated were subjected to PLA using antibodies against HA and GRP78. DAPI (*blue*) represents nuclei staining. *Yellow* indicates colocalization (Scale bars, 10 μm). *D*, similar to *A* except for IF staining for ACE2 (*red*) and GRP78 (*green*). (Scale bars, 20 μm *top row*, 5 μm *bottom row*). *E*, similar to *C* except VeroE6-ACE2 cells were subjected to PLA using anti-ACE2 and anti-GRP78 antibodies (Scale bars, 10 μm). *F*, PLA negative control groups. *Top row*, anti-GRP78 + Rabbit IgG isotype (GRP78/IgG); *middle row*, anti-ACE2 + Mouse IgG isotype (ACE2/IgG) and *bottom row*, Mouse IgG + Rabbit IgG (IgG/IgG) in permeabilized VeroE6-ACE2 cells. (Scale bar 40 μm).

Viruses, including SARS-CoV-2, usurp the host ER translational machinery to synthesize the viral proteins in massive quantities. Thus, as a major ER chaperone, GRP78 plays an essential role in viral protein synthesis and maturation (15, 17, 24, 25, 26). Confocal immunofluorescence (IF) microscopy of permeabilized cells expressing HA-Spike showed that it colocalized with endogenous GRP78 in the perinuclear region typical of the ER and in nonpermeabilized cells at the cell surface (Fig. 2*A*). The IF results were confirmed using the Proximity Ligation Assay (PLA), which reveals protein–protein interactions at distances <40 nm (Fig. 2, *B* and *C*). We note that in these proof-of-principle studies, the interaction between GRP78 and ectopically expressed HA-Spike at the cell surface could have originated from their interaction in the ER and not at the cell surface. By both confocal IF microscopy and PLA, colocalization between endogenous GRP78 and ACE2 was detected in the perinuclear region typical of the ER, and their colocalization was also observed on the cell surface (Fig. 2, *D*–*F*). Together, these studies suggest that GRP78 could serve as a foldase for SARS-2-S and ACE2 in the ER and act as a scaffold for SARS-2-S and ACE2 interaction on the cell surface. Recent studies showed that GRP78 deficiency could lead to a decrease in cell surface receptors such as CD109 and CD44 (21, 27). Next, we determined whether GRP78 deficiency would affect ACE2 expression. Interestingly, while knockdown of GRP78 by siRNA did not affect total ACE2 protein level under these experimental conditions, the level of csACE2 decreased markedly in parallel with a decrease in csGRP78, as determined by isolation of biotinylated cell surface proteins followed by western blot (Fig. 1*E*). As viral infection elicits ER stress, we further determined that upon treatment of VeroE6-ACE2 cells with an ER stress-inducing agent such as thapsigargin for 24 h, the level of total and csGRP78 increased while the level of total and csACE2 remained constant (Fig. 1*G*). Furthermore, knockdown of GRP78 led to the activation of markers of the unfolded protein response including p-eIF2α, ATF4, and CHOP, as well as cleavage of caspase 7 in these cells (Fig. 1*F*).

### Targeting GRP78 with monoclonal antibody reduces SARS-CoV-2 viral entry and infection

**Figure 3:**
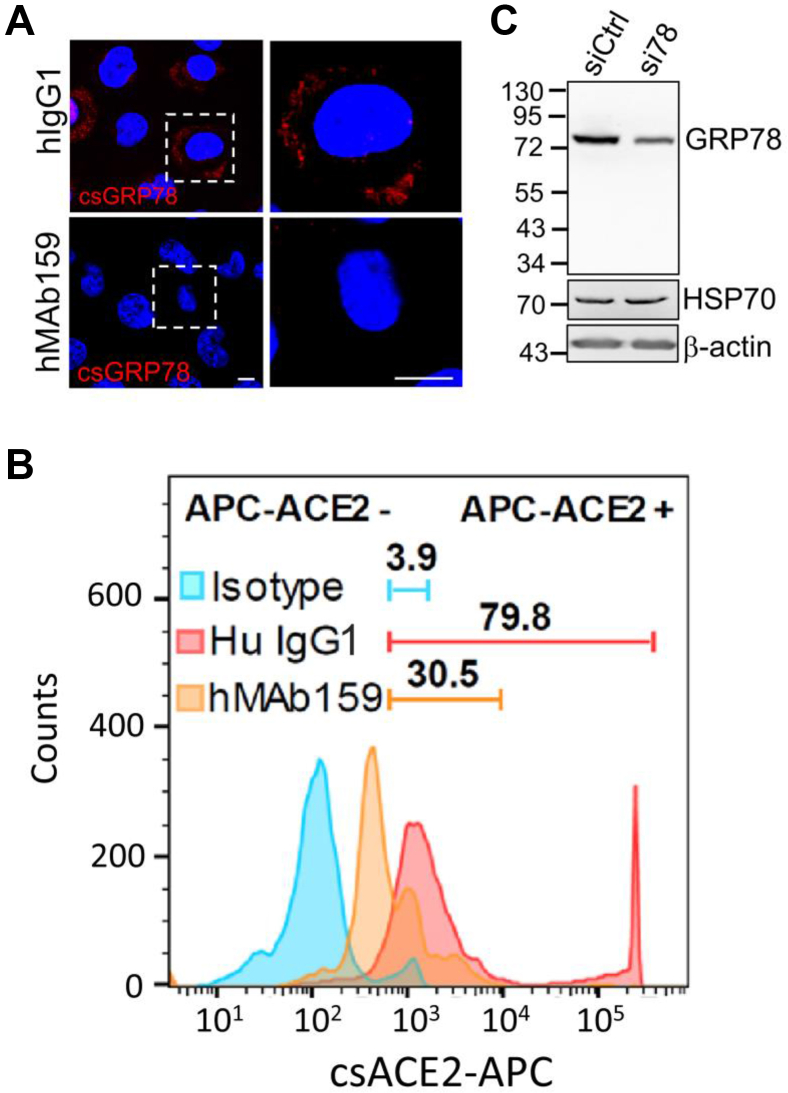
**Effect of hMAb159 treatment on cell surface forms of GRP78 and ACE2, viral entry, and infection.***A*, confocal IF images of nonpermeabilized H1299 cells treated with human IgG1 or humanized MAb159 (hMAb159) at 0.5 μg/ml for 2 h at 37 °C and probed with anti-GRP78 (*red*) antibody. DAPI (*blue*) indicates nuclei staining (Scale bars, 10 μm). *B*, flow cytometry analysis of cell surface ACE2 (csACE2) of the same cells treated with human IgG1 or hMAb159. Fluorescence intensity beyond the right border of negative control isotype was set as positive staining. The numbers indicate the percentage of csACE2 positive staining cells under each condition. *C*, western blot of H1299 cell lysates treated with control siRNA (siCtrl) or siRNA against GRP78 (si78) and probed with hMAb159 (*top panel*) or anti-HSP70 antibody (*middle panel*), with β-actin serving as loading control (*bottom panel*).

To test directly whether csGRP78 facilitates SARS-CoV-2 entry, we employed the human lung epithelial cell line H1299 and the vesicular stomatitis virus (VSV) pseudo particles bearing SARS-2-S as viral entry model system. To specifically target csGRP78 and deplete it from the cell surface, we utilized humanized MAb159 (hMAb159), a monoclonal antibody established to have high specificity and affinity against GRP78 with safe clinical profile in preclinical models (23). In H1299 cells, hMAb159 treatment led to reduced csGRP78 staining (Fig. 3*A*), consistent with the ability of MAb159, which recognizes the C-terminal region of GRP78 to cause GRP78 endocytosis and degradation established in other cell systems (23). Flow cytometry analysis of the same cells pretreated with either human IgG1 or hMAb159 showed that hMAb159 reduced both the number of cells expressing csACE2 and the level of csACE2 (Fig. 3*B*). In western blot analysis of H1299 cell lysate, hMAb159 only recognizes a single protein GRP78 and has no cross-reactivity with its closely related cytosolic homolog HSP70, reaffirming its high specificity for GRP78 (Fig. 3*C*).

**Figure 4:**
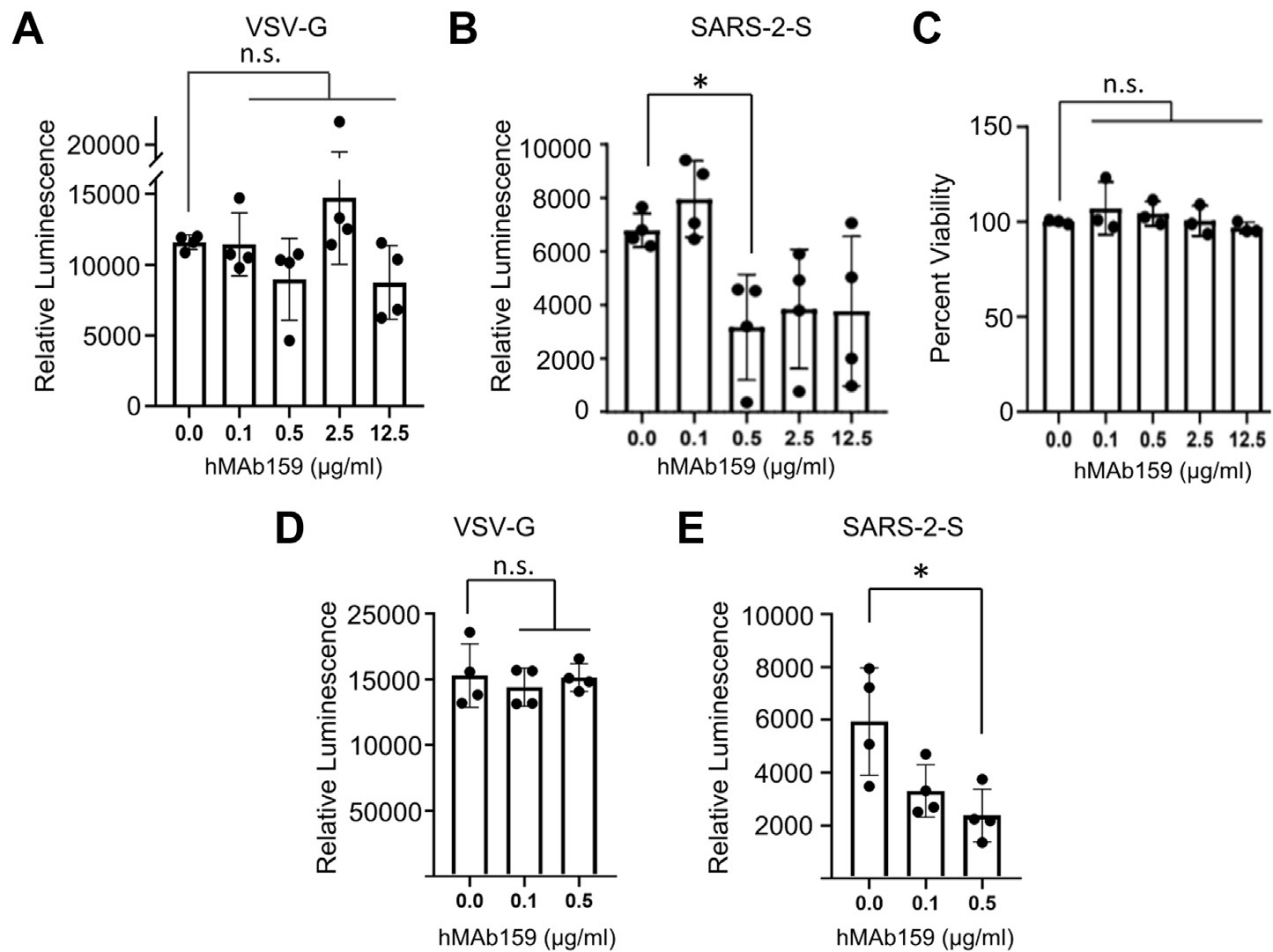
**Effect of hMAb159 in pseudotype viral entry assay.***A* and *B*, H1299 cells preincubated with indicated concentrations of hMAb159 2 h before transduction were subsequently inoculated with VSV pseudovirus harboring VSV-G or SARS-2-S surface receptor. At 16 h postinfection, relative luciferase activities were determined. Data are mean ± SD (n = 4). *C*, in parallel, after 18 h incubation of H1299 with hMAb159 at indicated concentrations, cell viabilities were measured *via* XTT assay. Data are mean ± SD (n = 3). *D* and *E*, Calu-3 cells were preincubated with the indicated concentrations of hMAb159 2 h before transduction, were subsequently inoculated with VSV pseudovirus harboring VSV-G or SARS-2-S surface receptor. At 16 h postinfection, relative luciferase activities were determined. Data are mean ± SD (n = 4). ∗ denotes *p* < 0.05 and n.s. denotes nonsignificant.

**Figure 5:**
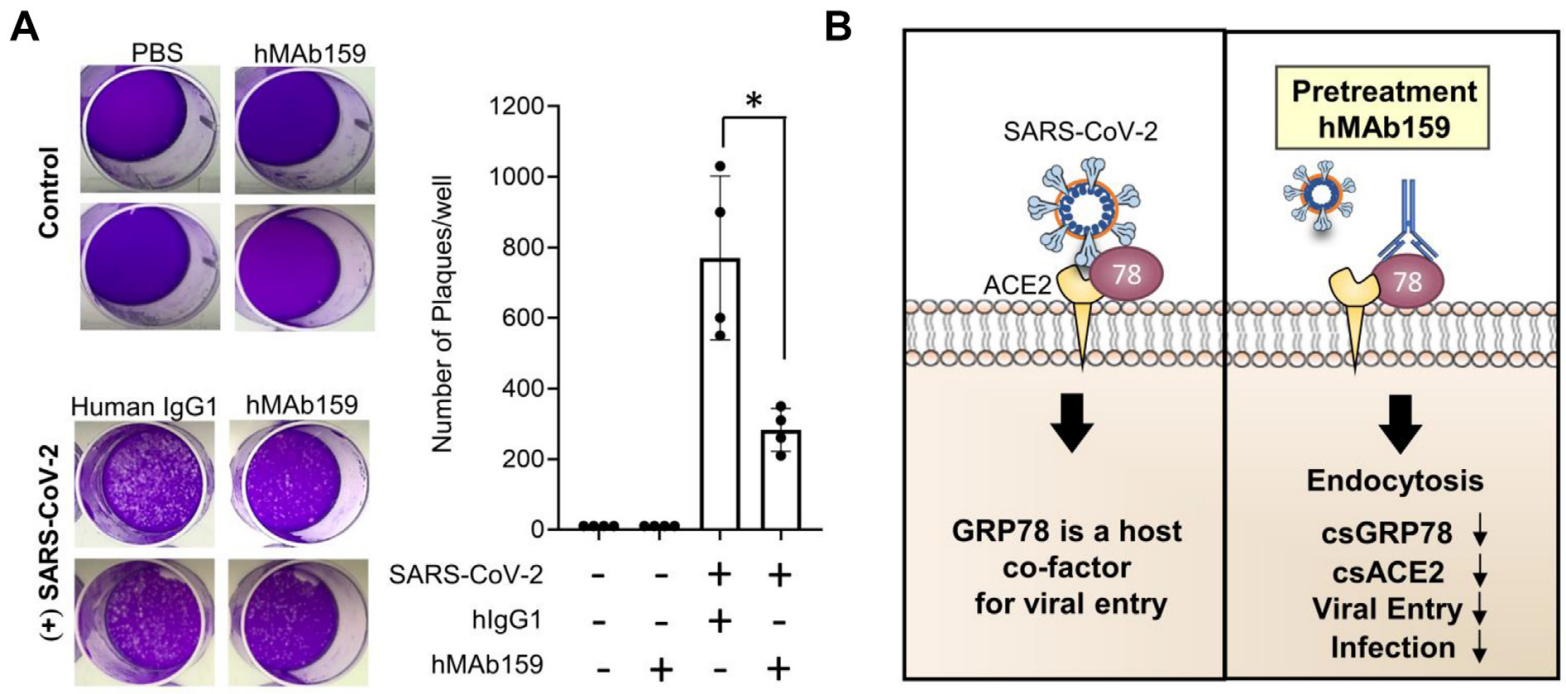
**hMAb159 pretreatment suppresses SARS-CoV-2 infection.***A*, plaque inhibition assay. VeroE6-ACE2 cells preincubated with 0.5 μg/ml hMAb159 or human IgG1 for 2 h were subsequently infected with live SARS-CoV-2 at MOI of 0.01. Viral replication was quantified by plaque assay. Data are mean ± SD (n = 4), ∗ denotes *p* < 0.05. *B*, summary diagram of our findings that through complex formation with the Spike protein of SARS-CoV-2 and the host receptor ACE2, GRP78 serves as a cofactor for viral entry (*left panel*) and that pretreatment with hMAb159 leads to reduction of the cell surface forms of GRP78 (csGRP78) and ACE2 (csACE2) and viral entry and infection are suppressed (*right panel*).

In viral entry assays, we observed that pretreatment with hMAb59 significantly reduced SARS-2-S-driven pseudovirus entry at concentration of 0.5 μg/ml but did not affect VSV-G-dependent entry into H1299 cells (Fig. 4, *A* and *B*) or cell viability, which excluded the possibility that the reduced SARS-CoV-2 entry was due to cytotoxicity caused by hMAb159 (Fig. 4*C*). Similarly, hMAb159 significantly reduced SARS-CoV-2 entry in another human lung epithelial cell line Calu-3 with no effect on VSV-G-dependent entry (Fig. 4, *D* and *E*). Furthermore, consistent with reduction of csACE2 and viral entry by hMAb159, VeroE6-ACE2 cells preincubated with hMAb159 prior to infection with live SARS-CoV-2 virus exhibited a significant decrease in the number of plaques compared with human IgG1 control (Fig. 5*A*). The key findings of this study are summarized in Figure 5*B*.

## Discussion

To our knowledge, our work provides the first experimental evidence that GRP78 is a direct binding partner of SARS-2-S in support of computer modeling predictions. Our results reveal that GRP78, in addition to potentially facilitating SARS-2-S binding to ACE2, is a novel regulator of ACE2 cell surface expression. Since total ACE2 protein level remains intact under these experimental conditions, this implies that GRP78 may be important for ACE2 trafficking, localization, and stability on the cell surface, and SARS-2-S production in the ER following viral infection, which awaits future investigation. Here, we define GRP78 as a new viral entry cofactor and a target for anti-SARS-CoV-2 intervention. hMAb159, which has high specificity and affinity for GRP78 and an established safe clinical profile in preclinical models, is ready for clinical development. This work uncovers its potential as a new therapy and use in combination with existing therapies could be further considered. Interestingly, a recent study showed GRP78 colocalizing with SARS-2-S following live virus infection and AR12, an inhibitor of chaperones including GRP78, suppressed SARS-CoV-2 infection (28). Collectively, these results suggest that targeting host auxiliary chaperones such as GRP78 required for viral entry and production could offer new strategies to suppress SARS-CoV-2 and possibly future coronavirus strains that may arise. It is also tempting to speculate that csGRP78 expression elevated in stressed organs and hypoxic endothelial cells (18, 29, 30) may contribute to higher viral entry and morbidity in COVID-19, which warrants further investigation. Finally, as a major ER chaperone and cell surface coreceptor, GRP78 has been implicated in the entry and production of a variety of viruses including Ebola virus where its re-emergence could pose serious public health concerns. Thus, instead of targeting individual viruses that are prone to mutations, targeting their critical auxiliary host chaperones such as GRP78 could have a broad-spectrum antiviral effect beyond SARS-CoV-2 with wide clinical implications.

## Experimental procedures

All methods are described in the Supplemental Experimental Procedures, including cell lines and culture conditions, expression vector construction and transfection, *in vitro* pull-down assay, immunoprecipitation, cell surface biotinylation, immunoblot analysis, flow cytometric analysis, immunofluorescent staining, proximity ligation assay, generation of VSV pseudotype and transduction experiments, quantification of cell viability, and plaque inhibition assay.

### Statistical analysis

All results are expressed as means. Error bars are reported as standard deviation. Differences between two group means were analyzed using a two-tailed unpaired Student’s *t*-test. A *p*-value less than 0.05 is statistically significant.

## Data availability

The authors confirm that the data supporting the findings of this study are available within the article.

## Supporting information

This article contains supporting information (20, 21, 23, 27, 31, 32).

## Conflict of interest

The authors declare that they have no conflicts of interest with the contents of this article.

## Supporting information

Supplemental Experimental Procedures
